# The prognostic impact of supraclavicular lymph node in N3-IIIB stage non-small cell lung cancer patients treated with definitive concurrent chemo-radiotherapy

**DOI:** 10.18632/oncotarget.16054

**Published:** 2017-03-09

**Authors:** Dongryul Oh, Yong Chan Ahn, Hee Chul Park, Do Hoon Lim, Jae Myoung Noh, Won Kyung Cho, Hongryull Pyo

**Affiliations:** ^1^ Department of Radiation Oncology, Samsung Medical Center, Sungkyunkwan University School of Medicine, Seoul, Korea

**Keywords:** radiotherapy, carcinoma, non-small cell lung cancer, chemoradiotherapy, supraclavicular lymph node metastasis

## Abstract

**Background:**

This study aimed to investigate the prognostic impact of supraclavicular lymph node (SCN) metastasis in patients who were treated with definitive chemoradiotherapy for N3-IIIB stage non-small cell lung cancer (NSCLC).

**Results:**

The 2- and 5-year overall survival (OS) rates were 57.3% and 35.7% in patients without SCN metastasis and 56.4% and 26.7% in patients with SCN metastasis, respectively. The median OS was 34 months in both groups. There was no significant difference in OS between the two groups (*p* = 0.679). The 2- and 5-year progression-free survival (PFS) rates were 24.1% and 12.6% in patients without SCN metastasis and 18.0% and 16.0% in patients with SCN metastasis, respectively. Patients without SCN metastasis had slightly longer median PFS (10 months vs. 8 months), but the difference was not statistically significant (*p* = 0.223). In multivariate analysis, SCN metastasis was not a significant factor for OS (*p* = 0.391) and PFS (*p* = 0.149).

**Materials and Methods:**

This retrospective analysis included 204 consecutive patients who were treated with chemoradiotherapy for N3-IIIB stage NSCLC between May 2003 and December 2012. A median RT dose of 66 Gy was administered over 6.5 weeks. Of these, 119 patients (58.3%) had SCN metastasis and 85 (41.7%) had another type of N3 disease: mediastinal N3 nodes in 84 patients (98.8%) and contralateral hilar node in one (1.2%). The patients were divided into two groups according to SCN metastasis.

**Conclusions:**

SCN metastasis does not compromise treatment outcomes compared to other mediastinal metastasis in the setting of definitive chemoradiotherapy.

## INTRODUCTION

Metastasis to supraclavicular lymph nodes (SCN) in non-small cell lung cancer (NSCLC) is an indicator of inoperable disease. Although surgical resection of SCN metastasis may be technically feasible, it has shown poor prognosis. [1, 2] In the American Joint Committee on Cancer (AJCC) staging system [1, 3] patients with metastasis to this node group are considered N3-IIIB stage. It is widely agreed that the current standard treatment for N3-IIIB stage NSCLC is concurrent chemoradiotherapy (CCRT) [2, 4]. Historically, in clinical trials to define the role of CCRT for stage III NSCLC, some groups have excluded SCN+ patients [5–7] while other groups included this population [8, 9]. Although several prospective randomized phase III trials demonstrated a survival benefit of CCRT for stage IIIB NSCLC [10, 11], there is still no clear evidence of whether patients with SCN+ NSCLC gain a comparable survival benefit to other mediastinal N3 patients when treated with CCRT. A recent study [12] showed that contralateral LN involvement, SCN involvement, and multilevel involvement did not decrease OS in patients with stage III NSCLC who were treated with definitive CCRT or RT, unlike the surgical series. We therefore performed a retrospective analysis to investigate the prognostic impact of SCN+ in patients who were treated with definitive CCRT for N3-IIIB stage NSCLC.

## RESULTS

### Patient characteristics

A total of 119 patients (58.3%) had SCN+ disease. Among them, SCN+ was confirmed pathologically by needle aspiration and biopsy in 85 patients (71.4%). Twenty-one patients (17.6%) had contralateral SCN+ and 12 (10.1%) had bilateral SCN+. Eighty-five patients had another type of N3 disease: mediastinal N3 nodes in 84 patients (98.8%) and contralateral hilar node in one (1.2%). The patients were divided into two groups according to their SCN+ status. There were no significant differences in clinical characteristics between the two groups except for use of intensity-modulated RT (IMRT) technique (Table 1). One hundred and sixty-seven patients (81.9%) were treated by three-dimensional conformal radiation therapy (3D-CRT) and 37 patients (18.1%) by IMRT. IMRT technique was used more frequently in SCN+ patients (*p* = 0.002). Forty-one patients (20.1%) received consolidation chemotherapy after the completion of CCRT.

**Table 1:** Characteristics of all patients according to the presence of supraclavicular lymph node (SCN) involvement

	Characteristics	Number of patients (%)		*p*-value
		SCN (−)	SCN (+)	
Age	< 60	38 (44.7)	57 (47.9)	0.652
	≥ 60	47 (55.3)	62 (52.1)	
Gender	Male	56 (65.9)	88 (73.9)	0.213
	Female	29 (34.1)	31 (26.1)	
Performance	ECOG 0	4 (4.7)	7 (5.9)	0.892
	ECOG 1	80 (94.1)	110 (92.4)	
	ECOG 2	1 (1.2)	2 (1.7)	
Weight loss^a^	Yes	10 (11.8)	16 (13.4)	0.723
	No	75 (88.2)	103 (86.6)	
T stage	T1	19 (22.4)	30 (25.2)	0.489
	T2	41 (48.2)	55 (46.2)	
	T3	11 (12.9)	18 (15.1)	
	T4	10 (11.8)	15 (12.6)	
	Tx	4 (4.7)	1 (0.8)	
Histology	Adenocarcinoma	51 (60.0)	67 (56.3)	0.307
	Squamous cell carcinoma	28 (32.9)	33 (27.7)	
	Large cell carcinoma	1 (1.2)	7 (5.9)	
	Adenosquamous cell carcinoma	1 (1.2)	1 (0.8)	
	NSCLC-NOS	4 (4.7)	11 (9.2)	
Primary tumor	Upper lobe	47 (55.3)	75 (63.3)	0.311
location^b^	Lower lobe	38 (44.7)	44 (37.0)	
RT technique	3D-CRT	78 (91.8)	89 (74.8)	0.002
	IMRT	7 (8.2)	30 (25.2)	
Consolidation CTx	Yes	14 (16.5)	27 (22.7)	0.275
	No	71 (83.5)	92 (77.3)	

^a^weight loss more than 5% within six months, ^b^Right middle lobe included in the upper lobe, SCN+: supraclavicular lymph node-positive, ECOG: Eastern Cooperative Oncology Group, NSCLC-NOS: non-small cell lung cancer-not otherwise specified, RT: radiation therapy, 3-dimensional conformal radiation therapy (3D-CRT), intensity-modulated RT (IMRT), CTx: chemotherapy.

### Survival

The 2- and 5-year overall survival (OS) rates of all patients were 56.8% and 31.6%, respectively. The median OS time was 34 months. The 2- and 5-year progression-free survival (PFS) rates of all patients were 21.2% and 14.1%, respectively, and the median PFS time was 9 months.

**Figure 1:**
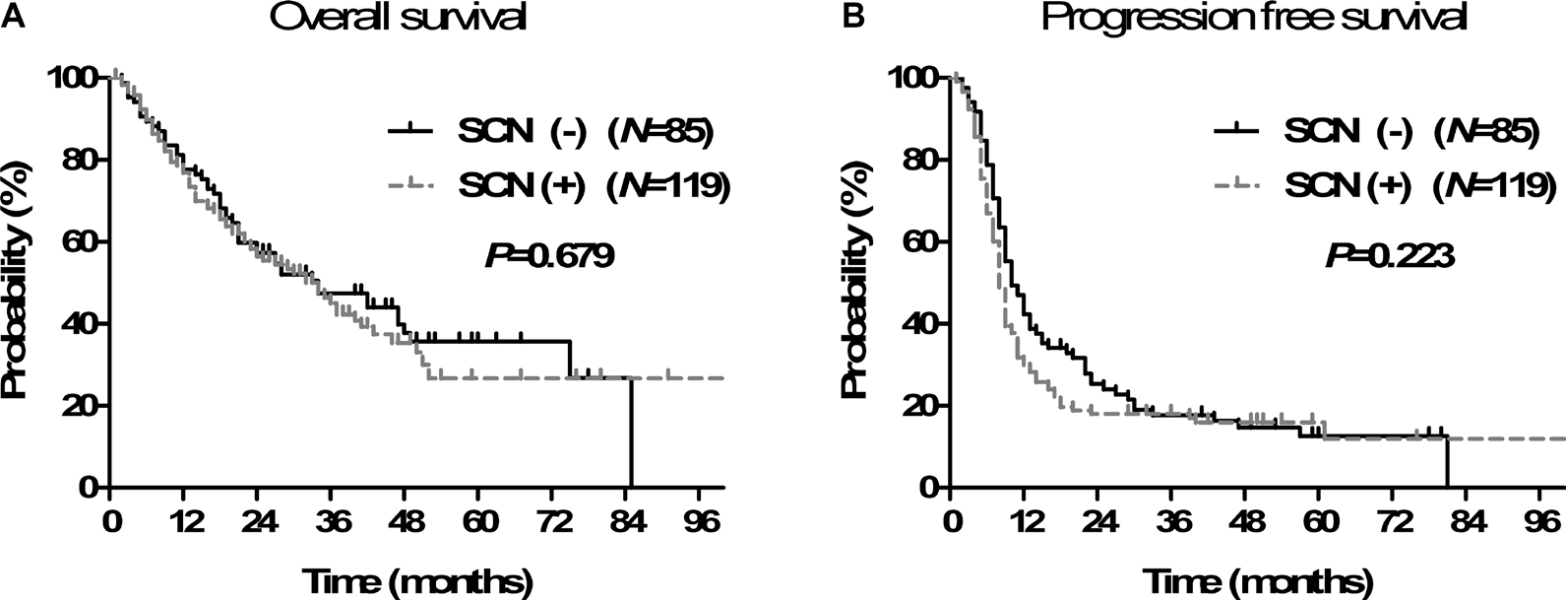
Overall survival (**A**) and progression-free survival (**B**) according to supraclavicular lymph node (SCN) involvement.

The treatment outcomes were compared according to SCN involvement. The 2- and 5-year OS was 57.3% and 35.7% in SCN- patients and 56.4% and 26.7% in SCN+ patients, respectively (Figure 1A). The median OS time was 34 months in both groups. There was no significant difference in OS between the two groups (*p* = 0.679). The 2- and 5-year PFS was 24.1% and 12.6% in SCN- patients and 18.0% and 16.0% in SCN+, respectively (Figure 1B). The SCN- patients had a slightly longer median PFS (10 months in SCN- vs. 8 months in SCN+ patients), but the difference was not statistically significant (*p* = 0.223).

### Pattern of failure

One hundred and forty-six patients (71.6%) experienced disease recurrence at the time of analysis. In analysis of the first site of recurrence, distant metastasis was the most common pattern of failure. Local recurrence occurred in 34 patients (16.7%), regional recurrence in 43 patients (21.1%), and distant metastases in 101 patients (49.5%). There was no difference in pattern of failure according to SCN+ status (Table 2).

**Table 2:** Pattern of first failures according to supraclavicular lymph node (SCN) involvement

Pattern of failures	Number of patients (%)			
	SCN (−) (*N* = 85)	SCN (+) (*N* = 119)	All patients (*N* = 204)	*p* value
Local	5 (5.9)	11 (9.2)	16 (7.8)	0.378
Local, Regional	5 (5.9)	4 (3.4)	9 (4.4)	0.387
Local, Distant	2 (2.4)	3 (2.5)	5 (2.5)	0.939
Regional	11 (12.9)	9 (7.6)	20 (9.8)	0.203
Regional, Distant	4 (4.7)	6 (5.0)	10 (4.9)	0.913
Distant	31 (36.5)	51 (42.9)	82 (40.2)	0.359
Local, Regional, Distant	2 (2.4)	2 (1.7)	4 (2.0)	0.733
Local, all	14 (16.5)	20 (16.8)	34 (16.7)	0.949
Regional, all	22 (25.9)	21 (17.6)	43 (21.1)	0.155
Distant, all	39 (45.9)	62 (52.1)	101 (49.5)	0.381
Total	60 (70.6)	86 (72.3)	146 (71.6)	

SCN+: supraclavicular lymph node-positive.

### Prognostic factors

Univariate analysis revealed no statistically significant factor for OS; SCN involvement (*p* = 0.679), age less than 60 years (*p* = 0.114), gender (*p* = 0.226), T1 to 2 stage (*p* = 0.067), histology of non-adenocarcinoma (*p* = 0.442), primary tumor location (*p* = 0.*9*74), IMRT technique (*p* = 0.181), and consolidation chemotherapy (*p* = 0.192) were not significant factors. Similarly, there was no statistically significant factor for OS in multivariate analysis. SCN involvement did not decrease the OS (*p* = 0.391, HR: 1.185 [95% CI: 0.804 to 1.747]) (Table 3).

**Table 3:** Multivariate analysis for overall survival (OS) and progression free survival (PFS)

Characteristics	OS		PFS	
	HR (95% CI)	*p* value	HR (95% CI)	*p* value
Age (< 60 years vs. ≥ 60 years)	0.777 (0.533–1.132)	0.189	1.015 (0.738–1.397)	0.925
Gender (male vs. female)	1.110 (0.707–1.741)	0.650	0.879 (0.608–1.271)	0.493
cT stage (cT1-2 vs. cT3-4)	0.676 (0.437–0.1044)	0.077	0.715 (0.490–0.044)	0.083
Histology (non-adenoca vs. adenoca)	0.933 (0.617–1.410)	0.741	0.644 (0.453–0.915)	0.014
Primary tumor location (upper vs. lower)	0.783 (0.515–1.192)	0.783	0.956 (0.671–1.362)	0.802
RT technique (IMRT vs. 3D-CRT)	0.631 (0.344–1.158)	0.137	1.185 (0.779–1.803)	0.428
Consolidation CTx (Yes vs. No)	0.760 (0.478–1.207)	0.244	0.575 (0.386–0.857)	0.007
SCN involvement (Yes vs. No)	1.185 (0.804–1.747)	0.391	1.269 (0.918–1.754)	0.149

^*^ Right middle lobe included in the upper lobe, adenoca: adenocarcinoma, RT: radiation therapy, 3D-CRT: 3-dimensional conformal radiation therapy, IMRT: intensity-modulated RT, CTx: chemotherapy, SCN: supraclavicular lymph node.

In univariate analysis for PFS, SCN involvement (*p* = 0.223), age less than 60 years (*p* = 0.899), gender (*p* = 0.178), T1 to 2 stage (*p* = 0.188), primary tumor location (*p* = 0.858) and IMRT technique (*p* = 0.081) were not significant factors for PFS. Consolidation chemotherapy (*p* = 0.027) was the only significant factor for better PFS and histology of non-adenocarcinoma (*p* = 0.058) was marginally significant. In multivariate analysis, histology of non-adenocarcinoma (*p* = 0.014, HR: 0.644 [95% CI: 0.453 to 0.915]) and consolidation chemotherapy (*p* = 0.007, HR: 0.575 [95% CI: 0.386 to 0.857]) were significant factors for better outcome. SCN involvement did not decrease the PFS (*p* = 0.149, HR: 1.269 [95% CI: 0.918 to 1.754]) (Table 3).

### Toxicity

Grade 3 or higher pneumonitis occurred in 7 (5.8%) of the SCN+ patients and 2 (2.4%) of the SCN- patients. Grade 3 or higher esophagitis occurred in 15 patients (12.6%) in the SCN+ group and 11 patients (12.9%) in the SCN- group. Skin toxicity occurred only in SCN+ patients; 15 patients (12.6%) experienced grade 2 skin toxicity.

## DISCUSSION

The international staging system for lung cancer was accepted in 1986 [13] and SCN was considered to be “regional”, as for other N3 lymph nodes. This was not based on the prognosis but because the treatment portal could encompass the SCN in the same field as the primary tumor and mediastinal lymph nodes [14]. The current study showed that the prognosis of SCN+ patients was comparable to that of SCN- N3 patients in the setting of definitive CCRT. This indicates that metastasis to SCN is also regional based on the prognosis.

The current standard treatment for N3 NSCLC is CCRT. However, it was unclear whether SCN+ patients could benefit from CCRT because the proportion of SCN+ patients was not reported in most randomized controlled studies for CCRT in stage III NSCLC patients [10, 11]. Furthermore, there have been no prospective randomized trials to investigate the role of CCRT in SCN+ stage IIIB NSCLC, and such trials may not be easy to perform because the proportion of SCN+ patients is relatively small. Thus, retrospective analyses may be the only way to find the appropriate answer to this question. In the current retrospective study, we showed that patients with SCN+ N3 stage NSCLC achieved results comparable to those with other mediastinal N3 stage disease when treated with CCRT. The median OS for SCN+ patients was 34 months, which was the same as for SCN- patients. The median PFS also showed no statistical difference between the two groups (10 months in SCN- vs. 8 months in SCN+ patients, *p* = 0.233). In 1999, Machtay et al. [15] retrospectively analyzed the treatment outcomes of 256 stage IIIB patients using data from the RTOG trials of CCRT for NSCLC. They also showed that the 47 SCN+ patients had similar outcomes to the 209 SCN- patients, with median OS of 16.2 months for SCN+ patients and 15.6 months for SCN- patients. The study by Machtay differs from our study in that they compared the outcomes of SCN+ patients with those of other stage IIIB patients, but not N3 patients, and they also included patients treated with sequential chemoradiotherapy. In their report, 67.0% of SCN- stage IIIB patients were T4N0-2 and 30% of them received sequential chemoradiotherapy. Although we aimed to include only N3 patients treated with CCRT, the SCN+ patients still showed a prognosis comparable to that of SCN- N3 patients.

The prognostic impact of nodal metastasis on survival has been demonstrated predominantly in surgical series. Since the possibility of adequate clearance is important for prognosis in surgical resection, nodal extent and location are significant factors for oncologic outcomes. In fact, the standard treatment for locally advanced stage III NSCLC is definitive CCRT. The prognostic impact of nodal extent and location in the setting of CCRT may be distinct from that in the setting of surgical resection because a homogenous radiation dose can be directed to involved sites using modern RT techniques. Annemarie et al. [12] demonstrated that contralateral LN involvement, SCN involvement, and multilevel involvement did not decrease OS in an analysis of 106 stage III patients who were treated with definitive CCRT or RT. We also showed that SCN metastasis was not associated with poor oncologic outcomes among N3 stage patients.

RT toxicity in SCN+ patients was comparable to that in SCN- patients. The incidence of grade 3 or higher pneumonitis or esophagitis was not higher in SCN+ patients treated with CCRT, although grade 2 dermatitis such as moist desquamation was more frequent. This toxicity was mostly confined to the skin folds of the lower neck because of the arm-up treatment position.

Our study has the limitation of selection bias inherent to a retrospective analysis based on data from a single institution. However, we included all consecutive patients treated with CCRT using 3D-CRT or IMRT between 2003 and 2012.

In summary, our data indicate that supraclavicular lymph node metastasis does not compromise treatment outcomes compared to other mediastinal N3 metastasis in the setting of definitive CCRT. A larger cohort study addressing the prognostic significance of nodal extent in the setting of definitive CCRT is warranted.

## MATERIALS AND METHODS

### Patients

A total of 204 patients who had N3-IIIB stage NSCLC and were treated with CCRT between May 2003 and December 2012 were consecutively included in the current retrospective analysis. This analysis was approved by our institutional review board. Pathologic confirmation of NSCLC was made in all patients by bronchoscopy or percutaneous needle aspiration and biopsy. The clinical stage was determined according to the 7th AJCC staging system. The diagnostic and staging workups included taking a complete history and physical examination, simple chest X-rays, contrast-enhanced chest CT scans that routinely covered the liver and adrenal glands, brain magnetic resonance imaging (MRI), bronchoscopic evaluation with biopsy or washing cytology, complete blood counts, routine urinalyses, and blood chemistry profiles. Abdominal ultrasonography and CT were optionally included when clinically indicated. ^18^F-deoxy-glucose positron emission tomography (FDG-PET) or PET/CT was performed in almost all patients (*N* = 198, 97.1%).

SCN+ was confirmed pathologically using needle aspiration and biopsy if the short axis of the SCN was more than 5 mm on contrast-enhanced CT or FDG-uptake of SCN was greater than that of surrounding tissue on PET/CT. If SCN was clinically palpable and definitely positive on contrast-enhanced CT or PET-CT, needle aspiration and biopsy was often omitted for the diagnosis of SCN+.

### RT

A median RT dose of 66 Gy (range, 40–70 Gy) in once-daily doses of 2 Gy was administered over 6.5 weeks using involved-field RT. The gross tumor volume (GTV) was delineated based on all available clinical information, including radiologic imaging, PET scan, and bronchoscopy. The clinical target volume (CTV) was delineated by a 5-mm margin extending in all directions from the GTV, and the margins were modified in accordance with adjacent organs if necessary. An additional 5- to 8-mm margin extension around the CTV was included to generate the planning target volume. The 3D-CRT technique was mostly used. Three or four beam arrangements were typically used to adequately cover the target volume and minimize the dose to normal tissues such as the lungs, spinal cord, and esophagus. Some patients were treated with IMRT technique.

### Chemotherapy

Most patients received platinum-based chemotherapy concurrently during the course of RT. The regimens were as follows: weekly docetaxel (25 mg/m^2^) plus cisplatin (25 mg/m^2^) (*N* = 142, 69.6%); weekly paclitaxel (50 mg/m^2^) plus cisplatin (25 mg/m^2^) (*N* = 48, 23.5%); weekly paclitaxel (50 mg/m^2^) plus carboplatin (AUC 1.5) (*N* = 3, 1.5%); etoposide (50 mg/m^2^) plus cisplatin (50 mg/m^2^) every 4 weeks (*N* = 6, 2.9%); or pemetrexed (500 mg/m^2^) plus cisplatin (75 mg/m^2^) every 3 weeks (*N* = 5, 2.5%). Some patients received consolidation chemotherapy after the completion of CCRT. The addition of consolidation chemotherapy was determined individually with consideration of the patient's preference and performance status, the medical oncologist's discretion, and the enrollment of clinical trials.

### Follow-up

A chest CT was performed to evaluate initial response 1 to 3 months after the completion of RT, and patients were followed every 3–4 months during the first 2 years and every 6 months thereafter using chest CT and/or PET/CT. Treatment-related toxicity was evaluated according to the Common Terminology Criteria for Adverse Events, version 3.0.

### Statistics

OS was calculated from the start date of treatment to the date of the last follow-up or death. PFS was calculated from the start date of treatment to the date of the first recurrence or death. Survival rates were determined by the Kaplan-Meier method. Univariate analysis via the log-rank test was performed to assess differences between the groups, and the Cox proportional hazards regression model was employed for multivariate analysis. The distribution of categorical variables was analyzed by the Chi-square test or Fisher's exact test. A two-sided *p-value* of 0.05 or less was considered statistically significant.

## Abbreviations

3D-CRT: Three-dimensional conformal radiation therapy
AJCC: American Joint Committee on Cancer
CCRT: concurrent chemoradiotherapy
IMRT: intensity-modulated radiation therapy
NSCLC: non-small cell lung cancer
SCN: supraclavicular lymph node
